# Inferior Pubic Rami Stress Fracture in a High School Sprinter: A Case Report

**DOI:** 10.1155/cro/9959554

**Published:** 2026-05-30

**Authors:** Tyler Janitz, Rutledge Feltel, Tyler Schmitz, Jeff Nadwodny, Daniel Montero, George Pujalte

**Affiliations:** ^1^ Department of Family Medicine, Department of Orthopedic Surgery, Division of Sports Medicine, Mayo Clinic Florida, Jacksonville, Florida, USA, mayoclinic.org; ^2^ Department of Orthopedics, Prisma Health Blue Ridge, Anderson, South Carolina, USA

## Abstract

**Background:**

Pubic rami stress fractures comprise 1%–2% of all stress fractures and are most often seen in military recruits and long‐distance athletes; they are less commonly reported in short distance athletes. We present an atypical inferior pubic rami stress fracture in a high school sprinter and discuss the current treatment standards along with broad clinical implications of the case.

**Case Presentation:**

A 15‐year‐old female high school track athlete presented to the sports medicine clinic with right hip pain after she felt a pop around her posterior right hip while running a 100‐m sprint approximately 4 weeks prior. The pain was associated with severe, nonradiating pain, and inability to run afterwards. She came with bilateral hip and pelvic X‐rays from an outside facility that were reported as normal. However, the overread of the images by the sports medicine provider revealed a nondisplaced, subacute right inferior pubic rami stress fracture. The patient was instructed to refrain from running for 3 months. Repeat imaging at 2‐ and 3‐months postinjury revealed progressive healing that clinically correlated with symptom improvement. She then entered a return‐to‐run program with physical therapy and had full return to sport without limitation at 5 months postinjury.

**Conclusion:**

It is important that sports medicine and orthopedic providers take a holistic approach, considering a broad differential that may be contributing to a patient′s presentation, even if the answer is a known fracture entity in a less frequently reported athletic subgroup.

## 1. Introduction

Pubic rami stress fractures comprise 1%–2% of all stress fractures and are most often seen in military recruits and long‐distance athletes; they are less commonly reported in short distance athletes [1, 2]. They most often occur due to the repetitive tensile force conducted by the adductor magnus muscle, causing an avulsion‐like fracture of fatigue [3]. Treatment typically involves rest with gradual return to sport or play over a 3–6‐month period [4].

## 2. Case Presentation

### 2.1. Presenting Complaint

A 15‐year‐old, healthy female track athlete presented to the sports medicine clinic with right hip pain.

### 2.2. History

Four weeks prior, she was practicing her 100‐m sprints when she felt a pop around her posterior right hip associated with severe pain and inability to run afterwards. She also had difficulty and pain with weight‐bearing activities including walking and sitting. Since the injury, her symptoms had slightly improved as she could jog with mild pain but could not sprint due to nonradiating anterior groin and buttock pain most present during the ground contact phase of her run. Of note, she reported regular menstrual cycles and a well‐rounded diet.

### 2.3. Physical Exam

On physical examination, she was healthy with an athletic build, and there was no swelling, erythema, or ecchymosis overlying the right hip. She rose from a seated position without difficulty. Squatting reproduced mild buttock pain on the right side. She had slightly decreased internal and external range of motion of the right hip. Tenderness to palpation of the right ischial tuberosity was present. The Stinchfield test was mildly provocative for right groin pain. Straight leg raise, FABER, and FADIR tests were all negative. Her lower extremity neurovascular exam was intact.

### 2.4. Diagnostic Assessment

**Figure 1:**
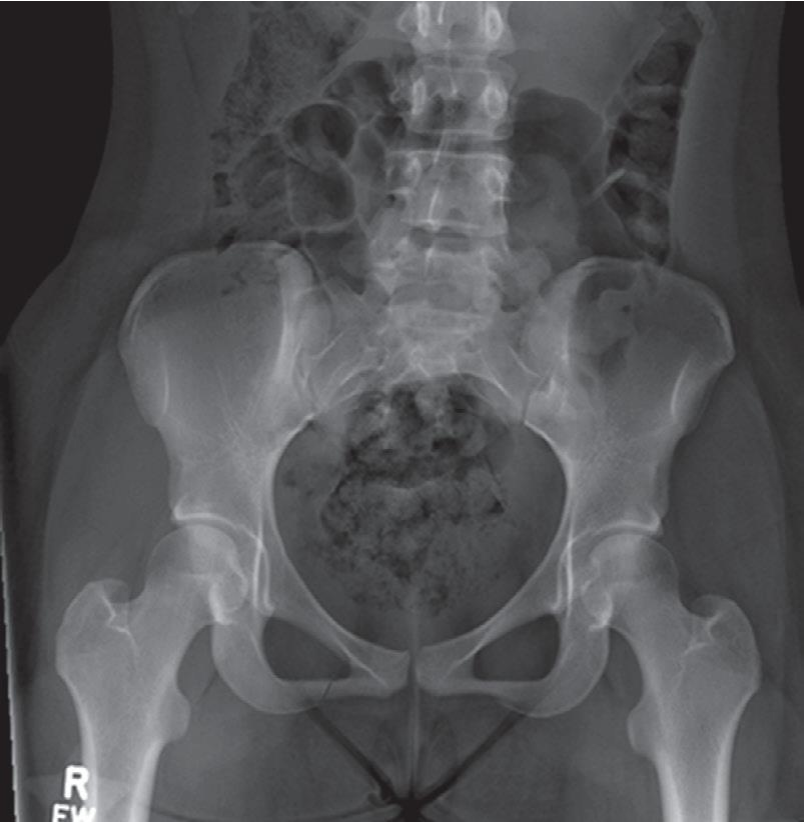
Pelvic X‐ray taken 2 weeks after initial injury showing a nondisplaced, subacute right inferior pubic rami fracture.

**Figure 2:**
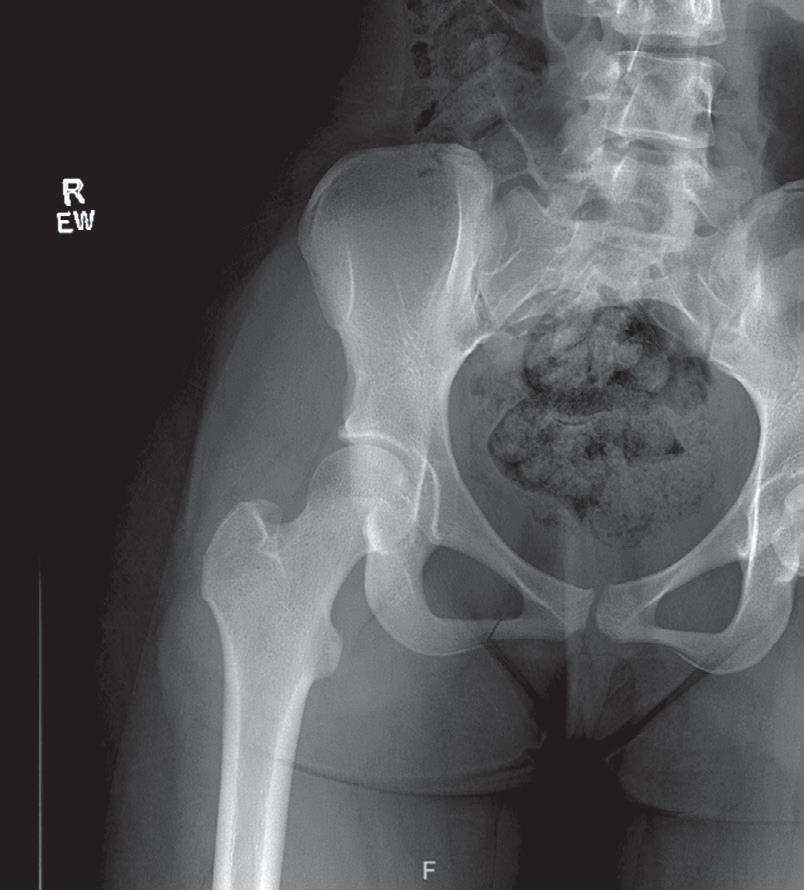
Another view of pelvic X‐ray taken 2 weeks after initial injury showing a nondisplaced, subacute right inferior pubic rami fracture.

The patient presented with outside bilateral hip and pelvic X‐rays obtained 2 weeks after injury that were read at an outside facility as unremarkable. However, a closer review of the images in the sports medicine clinic revealed a nondisplaced, subacute right inferior pubic rami stress fracture (Figures 1 and 2).

### 2.5. Follow‐Up and Outcome

**Figure 3:**
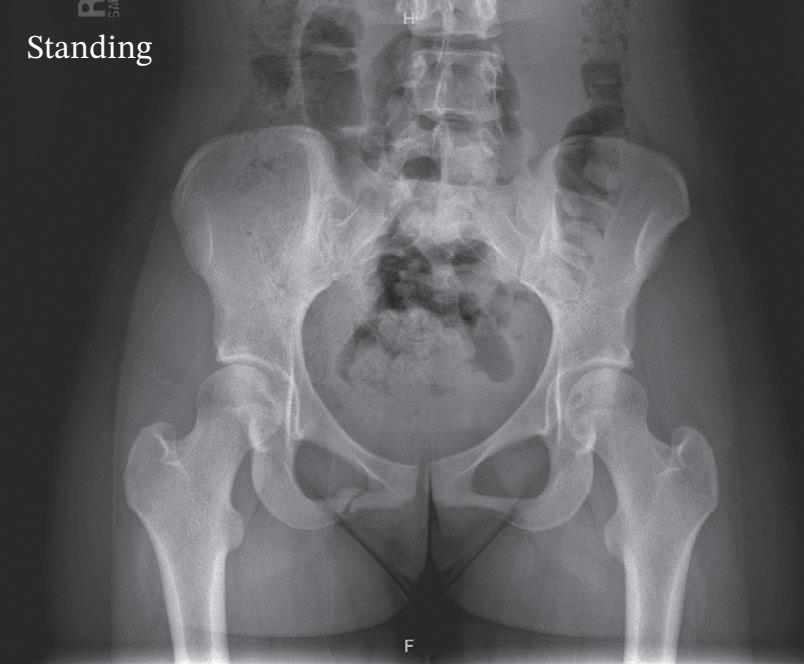
Interval X‐ray taken 2 months after initial injury showing healing of the nondisplaced fracture of the right inferior pubic ramus with callus formation.

**Figure 4:**
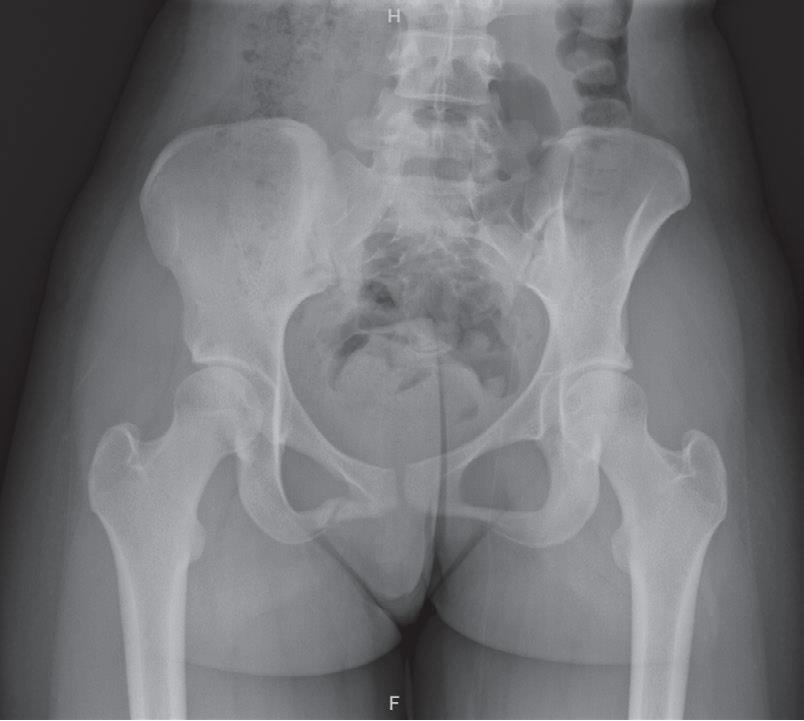
Interval X‐ray taken 3 months after initial injury showing progressive healing of the nondisplaced fracture of the right inferior pubic ramus.

The patient was instructed to avoid running for 3 months to allow for proper bone healing. The importance of nutrition and proper dietary intake was discussed as part of the treatment plan. Follow‐up X‐ray imaging at 2 months (Figure 3) and 3 months (Figure 4) postinjury revealed progressive healing that clinically correlated with symptom improvement.

At 4 months postinjury, she was referred to physical therapy for return‐to‐run program with good success. She returned to sport pain‐free without limitation 5 months after injury.

## 3. Discussion

Overall, there are very few pubic rami stress fractures reported in the medical literature as most osseous stress fractures tend to occur at the distal end of extremities where tensile forces against bone are the highest such as the metatarsal bones, tibia, and fibula [1, 3]. It is estimated that pelvic stress fractures comprise 1%–2% of stress fractures [1]. When present, they are mostly seen in military recruits and long‐distance athletes due to overuse and repetitive stress and are less commonly reported in short‐distance sprinters [2]. Pubic rami stress fractures occur mostly due to the repetitive tensile force conducted by the adductor magnus muscle, causing an avulsion‐like fracture of fatigue [5]. In sprinters specifically, the biomechanics of forceful hip flexion, powerful hip adduction during ground contact, and rapid eccentric loading during acceleration–deceleration movements with repetitive high magnitude loading generate high tensile and torque forces in the pubic tubercle and ramus areas [6]. The most common presenting complaint tends to be groin, inguinal, perineal, or buttock pain that is aggravated with weight bearing [1, 5]. Diagnosis is typically made by hip and pelvic X‐rays, but magnetic resonance imaging may be beneficial in inconclusive cases [5]. The differential diagnosis typically includes but is not limited to adductor strain, ischial apophysitis, femoral neck stress fracture, or pubic fracture. Treatment, especially in adolescents, typically involves rest with gradual return to sport or play over a 3–6‐month period with clinical and radiographic evidence of healing [4]. Failure to treat or return to full activity too quickly poses the risk of worsening fracture and improper bone healing [4].

## 4. Conclusion

Overall, it is important to include pubic rami stress fractures on the differential of groin or buttock pain. This patient′s presentation of inferior pubic rami fracture is less common in that she was a high school amateur sprinter. It is most likely that overuse and chronic tension of the lower extremity adductor muscles contributed to her fracture. Although proper sports nutrition and training were discussed with our patient, and she mentioned regular menstrual cycles with a well‐rounded diet, formal in depth screening was not completed due to lack of clinical suspicion. However, it is worth noting for future clinical practice that a more comprehensive nutrition evaluation with more detailed questioning such as a LEAF‐Q or BEDA‐Q questionnaire may have yielded valuable clinical information and produced a more complete assessment in this adolescent female athlete.

The patient came in with X‐rays that had been read as normal at an outside facility, but the provider overread the images and discovered the fracture that led to her appropriate diagnosis and treatment. Normal or unremarkable initial imaging is a well‐known diagnostic challenge for stress fractures, and clinicians should be cognizant of this potential clinical pitfall. Providers should read their own imaging and have a short threshold to escalate to more advanced imaging with MRI if clinical suspicion for stress fracture remains with inconclusive or normal radiographs. Of note, MRI was not completed and deemed unnecessary, as the radiographic overread at initial presentation clearly demonstrated the stress fracture at baseline that correlated with her clinical presentation and symptoms. Furthermore, for the current case, follow‐up imaging amidst rehabilitation and treatment showed fracture healing as her symptoms improved.

The importance of biomechanics in the sprinter cannot be undermined, and sports medicine providers should encourage and emphasize proper biomechanics and training regimens as a preventative measure against the development of stress fractures. As a final learning point, it is important that sports medicine and orthopedic providers take a holistic approach to patient and athlete care, considering a broad differential that may be contributing to a patient′s presentation, even if the answer is a known fracture entity in a less frequently reported athletic subgroup.

## Funding

No funding was received for this manuscript.

## Disclosure

This case was presented as a poster at the 8th Annual Mayo Clinic Sports Medicine for the Physician 2024 Conference in Orlando, Florida on March 8, 2024.

## Ethics Statement

The manuscript follows the CARE guidelines. The patient, who is over the age of 18 at time of publication, and guardian mother were both informed and provided consent for publication of clinical details and radiographic images included in this manuscript. IRB approval was not necessary for this manuscript. The authors have each contributed significantly to the production of the case.

## Conflicts of Interest

The authors declare no conflicts of interest.

## Data Availability

Data sharing is not applicable to this article as no new data were created or analyzed in this study.

## References

[1] Senisik S. and Ergun M. , Stress Fracture of the Pubic Ramus in a Female Long Distance Runner: A Case Report, Turkish Journal of Sports Medicine. (2017) 52, no. 2, 70–76, 10.5152/tjsm.2017.072.

[2] Yang B. K. , Yi S. R. , Ahn Y. J. , Im S. H. , and Park S. H. , Ischial Tuberosity Avulsion Stress Fracture After Short Period of Repetitive Training, Hip Pelvis. (2016) 28, no. 3, 187–190, 10.5371/hp.2016.28.3.187, 27777924. 27777924 PMC5067398

[3] Fredericson M. , Jennings F. , Beaulieu C. , and Matheson G. O. , Stress Fractures in Athletes, Topics in Magnetic Resonance Imaging. (2006) 17, no. 5, 309–325, 10.1097/RMR.0b013e3180421c8c, 2-s2.0-34247118297. 17414993

[4] Behrens S. B. , Deren M. E. , Matson A. , Fadale P. D. , and Monchik K. O. , Stress Fractures of the Pelvis and Legs in Athletes: A Review, Sports Health. (2013) 5, no. 2, 165–174, 10.1177/1941738112467423, 2-s2.0-84874573544, 24427386. 24427386 PMC3658382

[5] Dutton R. A. , Stress Fractures of the Hip and Pelvis, Clinical Sports Medicine. (2021) 40, no. 2, 363–374, 10.1016/j.csm.2020.11.007. 33673892

[6] Maloy W. , Merrigan B. , and Hulsopple C. D. , Groin Pain and Injuries: Evaluation and Management, American Family Physician. (2025) 111, no. 4, 337–343, 40238976. 40238976

